# Bitter taste receptors: Genes, evolution and health

**DOI:** 10.1093/emph/eoab031

**Published:** 2021-10-13

**Authors:** Stephen P Wooding, Vicente A Ramirez, Maik Behrens

**Affiliations:** 1 Department of Anthropology and Health Sciences Research Institute, University of California, Merced, CA, USA; 2 Department of Public Health, University of California, Merced, CA, USA; 3 Maik Behrens, Leibniz-Institute for Food Systems Biology at the Technical University of Munich, Freising, Germany

**Keywords:** bitter, molecular evolution, senses, taste, genetics, diet

## Abstract

Bitter taste perception plays vital roles in animal behavior and fitness. By signaling the presence of toxins in foods, particularly noxious defense compounds found in plants, it enables animals to avoid exposure. In vertebrates, bitter perception is initiated by TAS2Rs, a family of G protein-coupled receptors expressed on the surface of taste buds. There, oriented toward the interior of the mouth, they monitor the contents of foods, drinks and other substances as they are ingested. When bitter compounds are encountered, TAS2Rs respond by triggering neural pathways leading to sensation. The importance of this role placed TAS2Rs under selective pressures in the course of their evolution, leaving signatures in patterns of gene gain and loss, sequence polymorphism, and population structure consistent with vertebrates' diverse feeding ecologies. The protective value of bitter taste is reduced in modern humans because contemporary food supplies are safe and abundant. However, this is not always the case. Some crops, particularly in the developing world, retain surprisingly high toxicity and bitterness remains an important measure of safety. Bitter perception also shapes health through its influence on preference driven behaviors such as diet choice, alcohol intake and tobacco use. Further, allelic variation in *TAS2R*s is extensive, leading to individual differences in taste sensitivity that drive these behaviors, shaping susceptibility to disease. Thus, bitter taste perception occupies a critical intersection between ancient evolutionary processes and modern human health.

## Lay Summary

Bitter tastes warn animals about noxious substances in the environment, especially toxins found in plants. This has placed the genes controlling bitter taste under eons of pressure from natural selection. Signatures of these pressures remain in taste genes today, shaping food preferences, consumption, and health in animals and humans alike.

## INTRODUCTION

The diets of humans, their hominid ancestors and their primate relatives are dominated by plants, and for good reason [1]. Plants are abundant, productive and immobile, making them a potentially bountiful and accessible source of nutrition. However, plants are not as vulnerable as they seem. In a classic evolutionary arms race, they have adapted to herbivores, equipping themselves with an array of physical, reproductive and chemical defenses [2]. Substantial herbivore behavior and energy are dedicated to circumventing obstacles such as spines and thorns, shells and husks, synchronized fruiting and symbioses with animal defenders such as ants. Humans’ cognitive sophistication and adaptability make them uniquely successful at evading and even exploiting these protections, a talent catalyzing their dispersal from tropical Africa into virtually every ecosystem worldwide in the span of less than 75 000 years.

**Figure 1.**
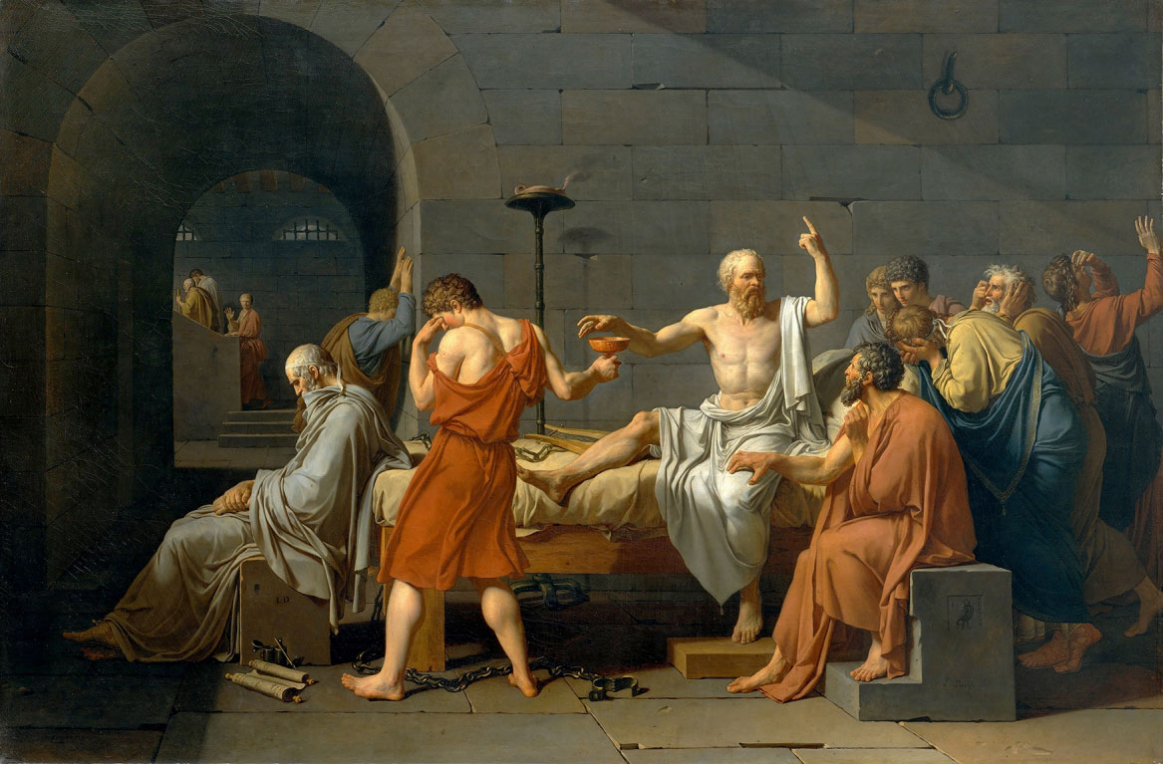
*The Death of Socrates*, by Jacques-Louis David (1787). Several lines of evidence suggest that Socrates was executed using extracts of hemlock (*Conium maculatum*) containing coniine and related alkaloids. Descriptions of Socrates's symptoms as he died to raise the possibility that additional compounds were included in the mixture but their identities remain unknown [5]

Perhaps the most formidable line of anti-herbivore defense in plants is their use of toxins. Nearly all plant species produce noxious compounds aimed at deterring predators [3, 4]. Some are notorious; alkaloids from poison hemlock were famously used to execute Socrates, and strychnine was used by an unknown assassin to kill Jane Stanford, co-founder of Stanford University (Fig. 1) [5]. However, the full repertoire of defense compounds used by plants reaches into the hundreds of thousands, with most receiving little notice because they are either not deadly or are rarely encountered [3]. For instance, urushiol, found in poison ivy, is irritating but not dangerous and abrin, found in rosary pea, is lethal at minute concentrations but encapsulated in easily recognized, undigestible seeds [6, 7]. Other notable examples among many include cyclopamine, an alkaloid in corn lily that interferes with embryogenesis, oleandrin, a cardiac glycoside in oleander that disrupts heartbeat, and cicutoxin, a polyacetylene in water hemlock that causes respiratory paralysis.

Herbivores have evolved a range of strategies for circumventing plants’ toxic defenses such as learned avoidance, metabolic denaturation and even symbiosis with microbiota [8, 9]. Among these, a particularly effective and widespread mechanism in vertebrates is bitter taste perception [10]. It has long been recognized that numerous plant toxins are perceived as bitter, and this provides a powerful means of reducing exposure. By signaling the presence of toxins in foods before they are fully ingested, bitter perception allows avoidance. Agriculture renders this role mostly redundant today. However, some crops retain toxicity despite domestication, and bitterness can be an important indicator of safety. Such is the case with cassava, a tropical crop containing neurotoxins that damage sensory, motor and cognitive function [11]. Other crops contain compounds that are bitter yet non-hazardous. This is the case with cruciferous vegetables, which contain the thyroid inhibitor goitrin [12, 13]. Goitrin is harmless in the quantities present in domesticated crucifers, yet its bitterness discourages consumption [14–17].

The ubiquity of bitter perception across vertebrates and its complex role in humans have raised questions about its evolution since the 1930s. Early studies comparing humans and chimpanzees concluded that natural selection has maintained variation in the two species, but inferences were limited by a lack of information about the specific genes involved [18, 19]. However, advancements have revealed the transduction pathways underlying bitter taste as well as the genes encoding them [20–27]. These are making possible the first high-resolution studies of molecular evolution in taste mechanisms, shedding light on the origins of bitter perception, variation in taste abilities within and among species, and their relationships with animal ecology and behavior [28, 29]. They are also providing new tools for dissecting the underpinnings of taste phenotypes, consumption behaviors and their nutritional consequences in humans. These lines of research continue to diversify and expand, painting an integrated portrait of connections between ancient evolutionary processes and modern human health.

## MECHANISMS OF BITTER PERCEPTION

**Figure 2.**
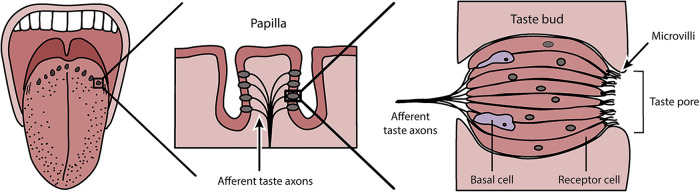
Tongue anatomy. Taste receptor cells are clustered in bundles beneath the surface of papillae, where they are exposed to the interior of the mouth through pores. TAS2Rs in the apical portion of the cells are poised to detect compounds in foods, smoke, pharmaceuticals and other ingested substances (©Casey Henley, CC BY-NC-SA 4.0 International License)

Bitter taste perception begins, of course in the mouth, whose surface is composed of epithelial tissue [30]. Embedded within the tissue on the tongue, soft palate and pharynx lie taste buds, clusters of receptor cells exposed to the mouth's interior through pores (Fig. 2). Each of the major taste qualities in humans (bitter, sweet, umami, sour and salty) is mediated by cells expressing corresponding receptor proteins, with salty and sour cells expressing ion channels and bitter, sweet and umami cells expressing G protein-coupled receptors (GPCRs) [31]. The receptors specifically responsible for initiating bitter perception were identified roughly 20 years ago and designated T2Rs, TAS2Rs, Tas2rs, or Tas2Rs, according to species (for convenience we refer to all using the human term, TAS2R, here) [24, 25, 32, 33]. This revealed their relationships with other GPCRs involved in environmental sensing including olfactory receptors (ORs) and opsins (RHO and OPNs), which operate through similar molecular mechanisms [34].

**Figure 3.**
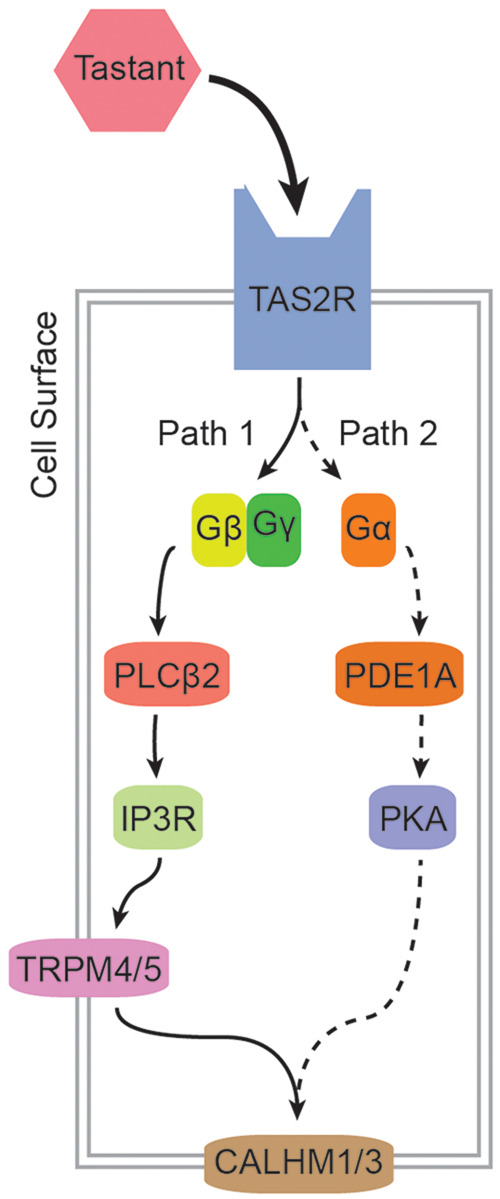
Bitter taste transduction cascade. TAS2Rs initiate the transduction process when exposed to compatible compounds. These interactions determine which substances are perceived as bitter and which are not, placing *TAS2R*s under selective pressures that vary according to diet

TAS2Rs localizes to the surface of bitter receptor cells, where they are exposed to chemicals in foods and other substances passing through the oral cavity. When stimulated by compatible compounds, TAS2Rs trigger a transduction cascade composed of two parallel pathways (Fig. 3) [35]. The first operates through the activation of phospholipase C β2 (PLCB2) by the β and γ subunits of TAS2Rs' cognate G protein. The second, which plays a modulatory role, operates through the activation of phosphodiesterase 1A (PDE1A) by the G protein's α subunit, α-gustducin. Downstream, ATP release is facilitated by activation of a calcium homeostasis modulator channel, CALHM1/3 [36–38]. Together these pathways depolarize the receptor cell, producing a signal conveyed to the central nervous system.

The discovery that TAS2Rs are bitter receptors was nearly simultaneous with the completion of the human genome project, which uncovered the complete set of genes encoding bitter transduction mechanisms. Remarkably, they included not one but 33 *TAS2R*s, with 25 functional loci and 8 pseudogenes occurring in clusters on chromosomes 5, 7 and 12 [24, 25, 39]. This makes the *TAS2R* family intermediate in size among sensory GPCR families such as opsins, of which there are four (*OPN*s and *RHO*), and olfactory receptors (*OR*s), of which there are hundreds [34]. *TAS2R*s were also found to be simple structurally, with each composed of a single exon ∼1kb in length (Table 1). The remaining components of the transduction cascade are represented by one gene each with the exception of phosphokinase A (PKA), a tetramer encoded by seven. The genes encoding transduction components are also more complex than *TAS2R*s, averaging 15 exons. Later findings in non-humans revealed that most vertebrates utilize the same system, with TAS2Rs initiating transductional cascades mediated by PLC β2.

**Table 1.** Human *TAS2R* genes, coordinates, transcript lengths and product sizes in the GRCh37 human genome map

Gene	GRCh 37 Coordinates	Transcript length	# Codons
*TAS2R1*	5: 9629109–9630463	1355	300
*TAS2R3*	7: 141463897–141464997	1101	317
*TAS2R4*	7: 141478242–141479235	994	300
*TAS2R5*	7: 141490017–141491166	1150	300
*TAS2R7*	12: 10954131–10955226	1096	319
*TAS2R8*	12: 10958650–10959892	1243	310
*TAS2R9*	12: 10961693–10962767	1075	313
*TAS2R10*	12: 10977916–10978957	1042	308
*TAS2R13*	12: 11060525–11062161	1637	304
*TAS2R14*	12: 11090005–11091862	1858	318
*TAS2R16*	7: 122634759–122635754	996	292
*TAS2R19*	12: 11174218–11175219	1002	300
*TAS2R20*	12: 11149094–11150474	1381	310
*TAS2R30*	12: 11285557–11287243	1687	320
*TAS2R31*	12: 11182986–11184006	1021	310
*TAS2R38*	7: 141672431–141673573	1143	334
*TAS2R39*	7: 142880512–142881528	1017	339
*TAS2R40*	7: 142919130–142920162	1033	324
*TAS2R41*	7: 143174966–143175889	924	308
*TAS2R42*	12: 11338599–11339543	945	315
*TAS2R43*	na	na	na
*TAS2R45*	na	na	na
*TAS2R46*	12: 11213964–11214893	930	310
*TAS2R50*	12: 11138512–11139511	1000	300
*TAS2R60*	7: 143140546–143141502	957	319
*TAS2R2P*	7: 12530721–12531630	910	∼303
*TAS2R12P*	12: 11047542–11048481	940	∼313
*TAS2R15P*	12: 11117024–11117951	928	∼309
*TAS2R18P*	12: 11311375–11312293	919	∼303
*TAS2R62P*	7: 143134127–143135066	940	∼313
*TAS2R63P*	12: 11200931–11201855	925	∼308
*TAS2R64P*	12: 11229915–11230841	927	∼309
*TAS2R67P*	12: 11332272–11333061	790	∼263

Coordinates of *TAS2R43* and *TAS2R45* are denoted na because their coordinates are ambiguous in GRCh37.

Like the hierarchical organization of the bitter transduction cascade, the expression of genes encoding the bitter transduction cascade highlights the specialization of TAS2Rs relative to other transduction components. Among the genes encoding the pathway, the most circumscribed expression pattern is that of TAS2Rs. They are highly expressed in bitter receptor cells in taste buds as well as in chemosensory cells in gut and bronchial smooth muscle, where they detect ingested and inhaled agonists [33, 40–42]. A critical feature of *TAS2R*s in taste bud cells is that they are coexpressed, with the average cell in humans expressing a random set of ∼5 to 10 of the 25 possible [40]. As a result, responsiveness to any particular compound can vary from cell to cell. Like TAS2Rs, α-gustducin is required for fully functional bitter perception. However, unlike TAS2Rs, it is also highly expressed in receptor cells responsive to sweet and savory compounds where it interacts with TAS1R receptors [43, 44]. Genes encoding other components of the pathway are expressed more broadly still, with some being highly generic to GPCR signaling processes and ubiquitously present across cell and tissue types [45–56].

While TAS2Rs are best known for their importance to perception, they play additional roles as well. They appear to be particularly important chemical sensors in the gut and airways, where they mediate responses to nutrients and inhaled substances [57–60]. For instance, stimulation of gut-expressed TAS2Rs triggers endocrine responses as well as processes such as gastric emptying [42]. Evidence that TAS2Rs can eavesdrop on bacterial quorum sensing activity in the gut suggests that they may also mediate responses to microbial conditions, which are both affected by and driven by health factors [59, 61]. Recent findings point to another function of TAS2Rs in the gut, the detection of parasites secreting-excreting TAS2R agonists [62]. Similar patterns are found in the airways, where TAS2Rs trigger responses to compounds originating from both inside and outside the body and likely mediate immune responses [59, 60].

## TAS2RS AND THEIR AGONISTS

**Figure 4.**
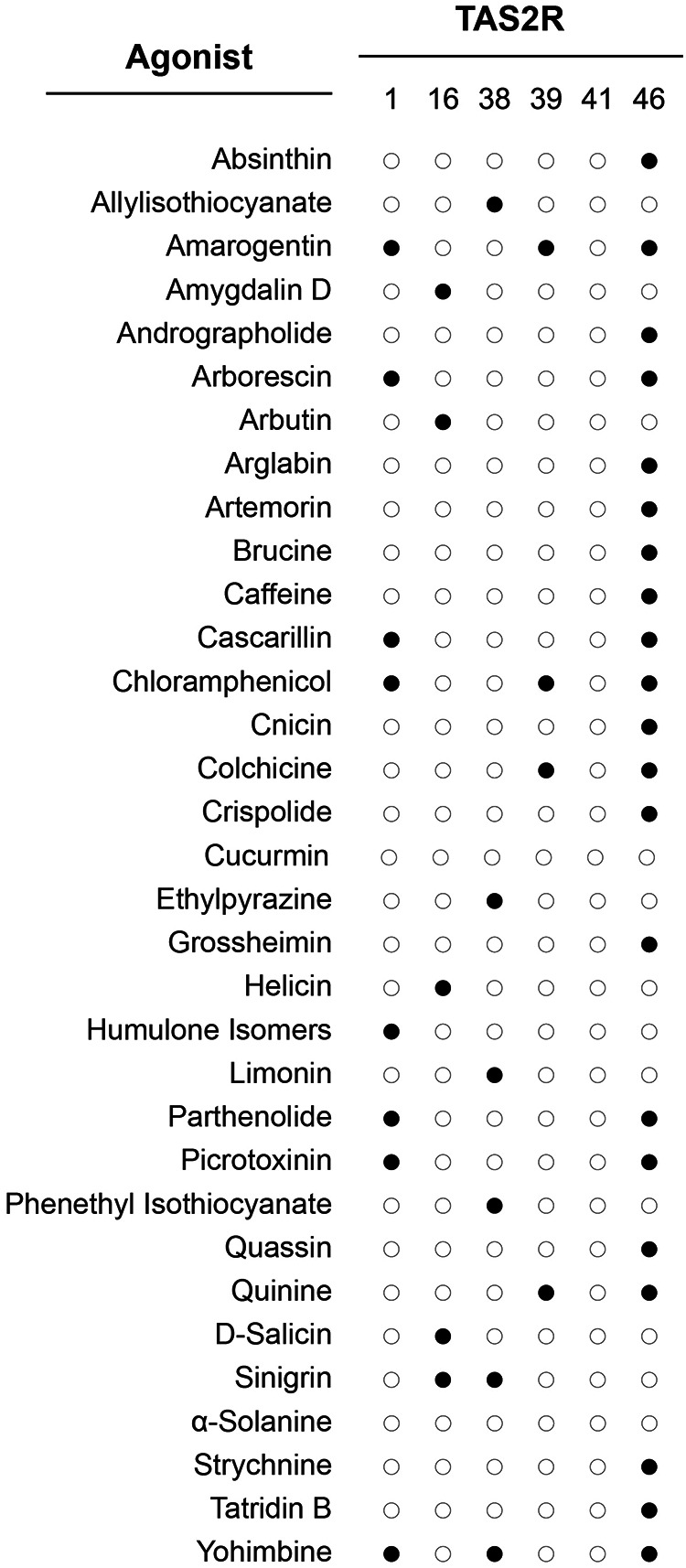
Example interactions between TAS2Rs and plant secondary compounds described by Meyerhof *et al*. [63]. Filled circles indicate activation, empty circles indicate no activation. Two key patterns are that most TAS2Rs are responsive to multiple compounds, and many compounds activate multiple TAS2Rs. These patterns extend across the TAS2R family in humans

The initial discovery that TAS2Rs are bitter taste receptors was based on their responses to a small number of well-defined compounds such as cycloheximide, denatonium and 6-*n*-propylthiouracil [32]. However, dozens of TAS2R agonists are now known, including numerous plant toxins as well as bitter yet non-toxic substances. In the most comprehensive study performed to date, Meyerhof *et al.* [63] used *in vitro* assays to determine the responses of all 25 human TAS2Rs to a library of 58 structurally diverse compounds, with an emphasis on toxins found in plants (Fig. 4). The results revealed that of the 25 tested TAS2Rs, 15 were activated by at least one of the 58 tested compounds. Conversely, of the 58 compounds, 43 were activated at least one TAS2R. In addition, across compounds tested, the number of responsive TAS2Rs ranged from 0 (no receptors for curcumin, digitonin, α-solanine and others were found) to 9 (quinine), and the number of compounds to which a given TAS2R was responsive ranged from 0 (TAS2R3, TAS2R5 and TAS2R9 and others responded to none of the 58) to 19 (TAS2R46). Moreover, when different TAS2Rs were responsive to the same compound they frequently exhibited different levels of sensitivity. These findings indicate that perception of most bitter compounds is not a simple function of compatibility between an agonist and a single receptor, it is a complex function of the full set of TAS2Rs to which the compound is agonistic, their individual patterns of response, and their variable expression across cells.

Surveys of receptor–agonist interactions in non-humans have revealed patterns similar to those in humans, with most TAS2Rs responding to multiple compounds and most compounds eliciting responses from multiple TAS2Rs [64]. Remarkably, many compounds agonistic to human TAS2Rs are also agonists of non-human TAS2Rs, and some compounds elicit responses from TAS2Rs in every species tested to date. This is surprising given the level of variability in diet among taxa, which would seem to predict that species carry receptors specifically targeting compounds encountered when foraging. A potential explanation for the overlap is that plants do not produce an unlimited diversity of secondary compounds. Most belong to families, such as alkaloids, terpenes and phenolics [3]. For instance, numerous plants produce glycosides, with the specific molecules varying across species but all sharing the chemical characteristics that make them glycosides. Hence, if a TAS2R is responsive to one compound it is likely responsive to others. Likewise, if a compound elicits responses from one TAS2R it is likely to elicit responses from TAS2Rs responsive to compounds with similar chemical structures. This phenomenon is documented for TAS2R16, which is broadly responsive to β-D-glucopyranosides, and TAS2R38, which is broadly responsive to isothiocyanates [17, 65–69]. Investigations using computational methods have yielded additional insights, suggesting that compounds with small globular structures tend to activate more TAS2Rs than do compounds with spacious and flat structures [70].

### The evolution of bitter receptors

The rapidly rising availability of whole-genome sequencing is providing, for the first time, perspectives on the evolution of *TAS2R*s as a family. One of the most remarkable revelations from comparisons across vertebrates is that *TAS2R*s are a relatively recent evolutionary innovation. Whereas they are present in the bony fishes, lobe-finned fishes and tetrapods, they are not found in cartilaginous fishes [64]. Thus, TAS2Rs must first have appeared in an aquatic species ∼430 million years ago [71]. This makes them the most recently derived of the chemosensory receptor families in vertebrates [64, 72, 73]. The pressures driving *TAS2R*s' earliest origins can only be speculated upon, but, notably, vascular plants underwent an explosive radiation ∼430 million years ago likened to the Cambrian explosion in animals [74, 75]. The coincidence suggests that the origin of *TAS2R*s may mark a pivotal transition in plant–herbivore interactions. However, there are compelling alternatives. In particular, evidence that TAS2Rs play roles outside of taste, especially nutrient sensing in the gut, suggests that TAS2Rs might have evolved to play those roles first with their function as taste receptors coming later.

**Figure 5.**
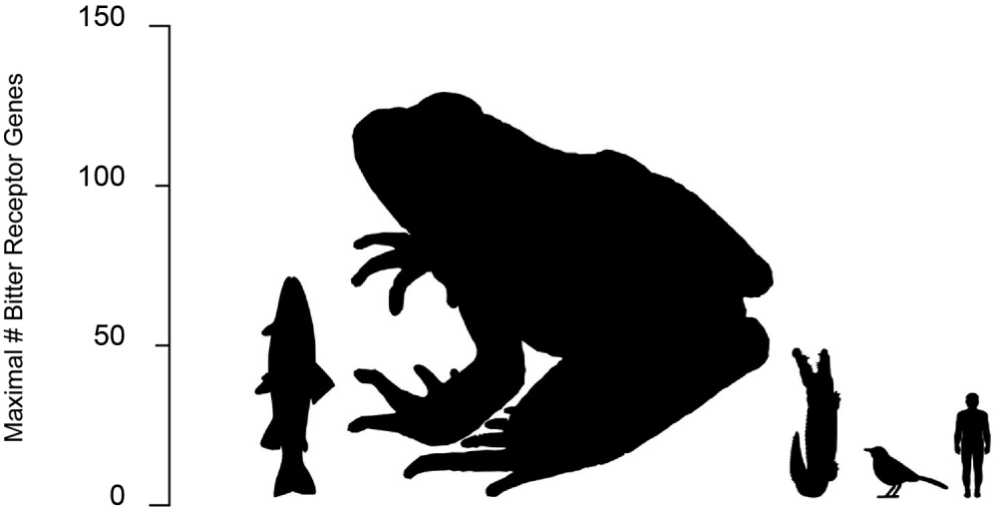
Size of bitter taste receptor gene repertoires in bony fish, amphibia, reptilia, aves and mammalia. Gene count is proportional to image height. Across species studied to date, the number ranges from 0 (in some marine mammals and birds) to 136 (in frogs). The large number of TAS2Rs in frogs may be due to their dietary reliance on insects, which often sequester secondary metabolites from consumed plants

Following its initial emergence, the *TAS2R* family diversified both within and among taxa. The estimated size of *TAS2R* repertoires continues to be refined as genome assemblies increase in precision, and trends are already evident (Fig. 5). The most conspicuous aspect of diversification is in the number of *TAS2R* loci in species’ genomes, which varies substantially. This is exemplified in fishes, in which gene counts range from one in Amazon molly (*Poecilia formosa*) to 74 in coelacanth (*Latimeria chalumnae*) [76–83]. However, most fishes carry an intermediate number, such as the 6 in Atlantic cod (*Gadus morhua*), 7 in zebra danio (*Danio rerio*) and 21 in blind cave fish (*Astyanax mexicanus*) [82]. Fish genomes also contain *TAS2R* pseudogenes. One is found in the spotted green puffer fish (*Tetraodon nigroviridis*) and three are found in *A. mexicanus* [82]. The presence of multiple *TAS2R*s in most species in conjunction with the presence of both functional and non-functional loci suggest that birth-death processes are a key aspect of *TAS2R* evolution, although little is currently known about them.

**Figure 6.**
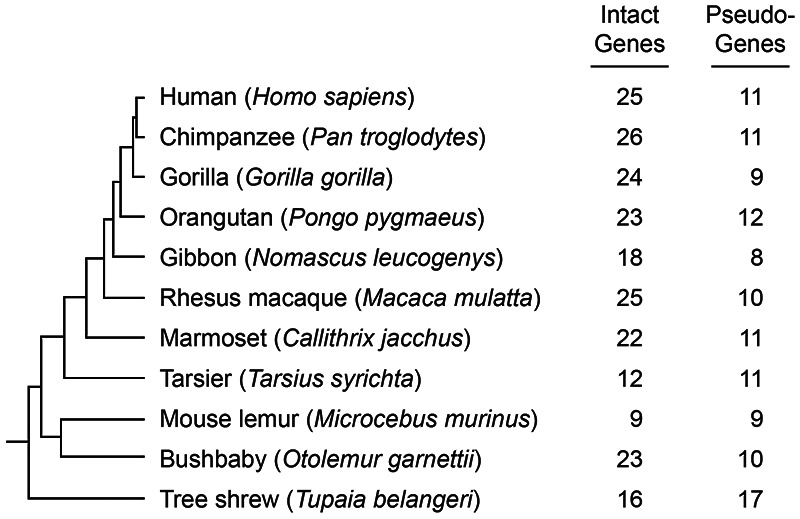
Intact genes and pseudogenes in primates. All primates studied to date harbor both intact genes and pseudogenes. However, their numbers vary even across closely related taxa, such as humans and chimpanzees. This suggests that gene birth–death processes are an important mechanism of adaptation to toxin exposure. Redrawn from Ref. [79]

Variation in the number of *TAS2R* genes occurs outside the fishes, as well. Among amphibians, cecilians have the smallest number (18–28) and anurans the largest of any recorded vertebrate (50–136) [76–79, 81, 84, 85]. Numbers in axolotl range from 35 to 49 depending on the study [76, 85]. The sauria, a highly heterogeneous taxon that includes the crocodilians, lizards, snakes and birds, are similarly variable. Snakes have low numbers (0–2) whereas crocodiles and turtles have more (5–9 and 5–11, respectively), and lizards have 36–50, making them comparable to many fishes [76, 77, 79, 84, 86, 87]. *TAS2R* repertoires in birds, which are highly diverse ecologically, are highly diverse as well. Strikingly, a survey of five penguin species revealed that none bear functional *TAS2R*s yet other avians such as zebra finch (*Taeniopygia guttata*) and white-throated sparrow (*Zonotrichia albicollis*) do have them, hosting 7 and 17, respectively [88]. Mammals have intermediate numbers of *TAS2R*s compared to other taxa, such as the 7 in platypus, 16–27 in marsupials and ∼35 in rodents [28, 79]. However, like penguins, the cetacea lack or nearly lack *TAS2R*s altogether, with 0 or 1 depending on the species [89, 90]. Most higher primates have roughly 25 intact *TAS2R*s and 10 pseudogenes (Fig. 6). The lowest number is found in gibbon (*Nomascus leucogenys*), which possesses 18 intact and 8 pseudogenes. Humans' closest relatives, chimpanzee, gorilla and orangutan possess 26, 24 and 23 intact loci, respectively.

While toxins are a threat to any animal eating plants, species are not equally vulnerable. For instance, carnivores rely minimally on plants and have little need to manage exposure. In contrast, leaf eating monkeys are constantly exposed and must adapt accordingly. This suggests that *TAS2R*s have evolved under different selective pressures in different species. Evidence for such differences is reflected in the size of *TAS2R* repertoires, which correlate with the proportion of species' diets composed of plants [79, 91]. The most conspicuous trend is that repertoires are larger in herbivores than in carnivores. The most extreme case, the complete absence of *TAS2R*s, is found in obligate carnivores that gulp rather than chew their food including whales, penguins and pinnipeds [79, 89, 91–93] (Hawaiian monk seal genome assembly neoSch1, 2017). Explanations for the opposite end of the repertoire size spectrum, in anurans, are less obvious. However, it may relate to their being insectivores. Many insects sequester secondary compounds from plants in their diets, coopting them for use in their own defense systems [94]. Examples abound, with the classic being monarch butterflies, which sequester cardenolides found in their food plant, milkweed. Insects' use of plant toxins suggests that even though they are not herbivores anurans encounter a broad spectrum of phytotoxins indirectly and benefit from being able to detect overly hazardous prey.

Although selective pressures on taste perception are the most obvious potential driver of diversification in *TAS2R*s, they are probably not the only driver. The extraoral roles of TAS2Rs point to pressures arising from their other functions, as well. For instance, because bitter perception shapes diet, which in turn shapes the gut microbiome, selective pressures on *TAS2R*s could originate from both roles simultaneously. Likewise, ambient air conditions vary from environment to environment, which could exert pressures on *TAS2R*s mediating respiratory responses. Thus, selective pressures on *TAS2R*s likely arise from their diverse roles in multiple systems, not solely from their role in perception.

### Population genetics

*TAS2R*s exhibit variability within species as well as between them. The earliest evidence for intraspecific variation was reported in 1932 by Fox [66], who observed that PTC sensitivity varies profoundly among individuals in most populations. Blakeslee [95] confirmed shortly thereafter that PTC perception is indeed heritable and is best explained by the presence of dominant and recessive alleles of a single gene, although the specific gene involved would remain unknown for nearly 70 years. These went on to be recognized as the famous PTC “taster” and “non-taster” alleles. The identity of the locus underlying PTC sensitivity was finally determined by Kim *et al.* [96], who discovered that two alleles of *TAS2R38* explain 50–80% of variance in the trait. Further, the two alleles differed by three amino acid changes, which often alter receptor function. And, as predicted by their amino acid differences, the two alleles exhibit divergent responses to PTC *in vitro* [97], corresponding to the long hypothesized taster and non-taster types. The finding that several amino acid variants occur in *TAS2R38*, along with the discovery that humans carry not one but 25 *TAS2R* loci, suggested that variation in *TAS2R*s and their associated phenotypes could be far reaching.

Owing to its early discovery and role in shaping a classic phenotype, *TAS2R38* is the most intensively investigated and best-understood *TAS2R* from a population genetic standpoint. In the first study aimed at documenting global sequence variation at *TAS2R38*, Wooding *et al.* [98] identified five variable amino acid positions in 165 subjects from Africa, Asia, Europe and North America, and they were configured into seven alleles. Notably, the canonical taster and non-taster alleles mapped by Kim *et al.* [96] were present at roughly equal frequencies and jointly accounted for 96% of the sample, with the remaining five accounting for just 4%. These findings confirmed that *TAS2R38* harbors a number of amino acid substitutions that might affect taste phenotypes but diversity worldwide is dominated by the three distinguishing the classic taster and non-taster variants. In the largest study of *TAS2R38* to date, Risso *et al.* [99] examined >5500 worldwide subjects and reported findings congruent with previous results. Twenty-four alleles were present, with the taster and non-taster variants distributed worldwide and a third common in Africa but rare elsewhere.

Insights into the population genetics of *TAS2R*s are no longer limited to *TAS2R38*. It is now known that allelic variation is abundant across the *TAS2R* family as a whole. In a study of all 25 *TAS2R*s in 55 subjects, Kim *et al.* [100] identified 144 nucleotide changes with a range from 1 (in *TAS2R13*) to 12 (in *TAS2R48*), 108 (75%) of which result in amino acid changes. In a survey of all *TAS2R*s across 22 subjects, Wang *et al*. [101] obtained similar results, observing 105 changes with a range of 0 (*TAS2R7*) to 12 (*TAS2R49*), 72 (70%) of which result in amino acid changes. These results demonstrate that all *TAS2R*s harbor variation likely to shape perception, and it is distributed widely across populations.

Studies of natural selection in additional *TAS2R*s further illuminate the evolutionary complexity of bitter perception. An instructive example is TAS2R16, which is responsive to glucopyranosides [102, 103]. Like *TAS2R38*, *TAS2R16* is represented by three primary alleles, which are defined by two amino acid changes. Also like *TAS2R38*, *TAS2R16* harbors two globally distributed alleles, along with a third one common in Africans but rare in non-Africans. However, *TAS2R16* contains a greater proportion of low-frequency nucleotide variants than does *TAS2R38*, a pattern expected under positive selection, not balancing selection [102, 103]. In addition, within Africa, some human alleles are unexpectedly similar to those of chimpanzees, which is indicative of strong purifying selection [102]. This suggests that reduced ability to perceive glucopyranosides is for some reason favored there. A speculative explanation offered by Soranzo *et al.* [103] is that low sensitivity to glucopyranosides could be favored if it results in greater consumption of them, which is thought to protect against malaria. However, this hypothesis was not supported by the results of Campbell *et al.* [102], who found no correlation between malaria prevalence and the frequency of the proposed protective allele. These findings reiterate the trends in *TAS2R38*, that selection on *TAS2R*s appears to be relaxed for some haplotypes but not others, and in some populations but not others. Similar complexity likely spans the rest of the *TAS2R* family, but it remains to be investigated.

### Health importance of bitter taste

Contemporary human diets rely principally on crops domesticated in the last 10 000 years, which were selectively bred to maximize productivity while minimizing the hazards of consumption [104]. Many are derived from ancestors that had low toxicity such as grasses, and selective breeding mainly improved calorie content. Such crops include rice, corn and wheat, which originated from species that were not toxic but were laborious to gather and process. However, some crops do originate from toxic predecessors. The most familiar example is potato, whose wild varieties produce alkaloids that render them inedible. Other examples include squashes, whose wild forms produce cucurbitacin, and beans, which produce phytohemagglutinin. Domestication of these species reduced toxicity to levels safe to consume with little preparation. However, plant toxins do remain in human diets, in some cases at high levels, and their bitterness is an important gauge of food safety.

**Figure 7.**
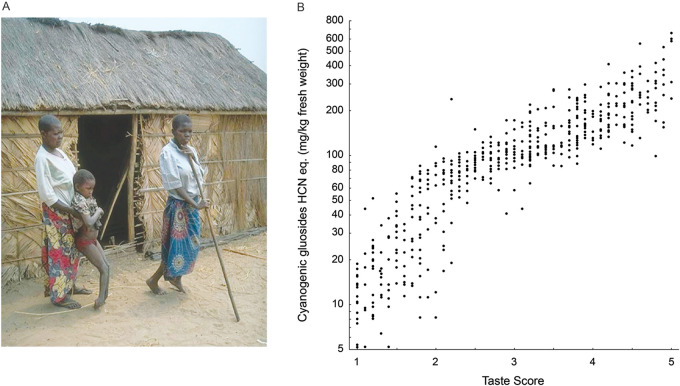
Cassava toxicity and konzo. (A) Konzo patients in the Democratic Republic of Congo. Note foot position of child and crutch used by an adult. Photograph courtesy of Dr Thorkild Tylleskär, University of Bergen. (B) Plot of cyanogenic glucoside content and perceived bitterness of samples tasted by farmers in Malawi. Higher taste score indicates higher perceived bitterness. Redrawn from Ref. [112]

The model example of bitter taste’s significance to food safety and disease involves cassava (*Manihot esculenta*). Though poorly known in industrialized countries, cassava is a major global crop. It ranks seventh in tonnage produced among crops worldwide and is a staple source of calories for ∼500 million people throughout the tropics [105]. However, cassava’s wide use belies a threat—it utilizes a highly toxic anti-herbivore defense system. When macerated, as by chewing, cassava releases neurotoxic nitriles and cyanide when an enzyme-substrate pair produced by the plant, linamarase and linamarin, react [11, 106]. Cassava's toxicity is valuable from an agricultural standpoint because it allows cultivation without pesticides or the manual labor needed to manage more delicate crops, reducing work and financial burden. However, cassava’s defense system is toxic to humans as well as pests. High dose exposures lead to quick death, and chronic exposures lead to nerve damage permanently impairing dexterity, locomotion and cognition [107]. Poisoning is so common in some regions of Africa that the resulting syndrome has a name, konzo (bound legs, in the Yaka language) (Fig. 7). Konzo is commonly found at frequencies ∼1–3% in affected areas and has been documented at frequencies >15% in extreme cases [108–111].

Cassava's toxicity can be eliminated prior to consumption through grinding and shredding, which drive its reactions to completion rapidly, or by peeling and soaking or drying, which do so more slowly. However, these processes are time consuming and energy intensive. This imposes countervailing pressures on growers, who must aim for toxicity high enough to protect the crop but low enough to avoid unnecessary labor removing it. Bitter taste helps resolve the conflict. Cassava cultivars vary in bitterness, and bitterness predicts toxicity [112]. Moreover, cassava’s bitterness is imparted by linamarin, the metabolic precursor to cassava's toxins, so taste and toxicity are directly connected [113]. Experienced growers are experts at assessing the toxicity of their crops based on taste, which they use for selecting which cultivars to grow and anticipating the effort required to process them (Fig. 7). Bitterness can also be used to monitor the level of residual toxicity as cassava is processed, ensuring the end product is safe to eat.

A second example of connections between taste, toxicity and health involves the traditional cultivation of potato (*Solanum tuberosum*) in Andean populations [114]. Potatoes are in the plant family *Solanaceae*, which is noted for its many toxic members including nightshade (*Atropa* spp.), jimsonweed (*Datura* spp.) and tobacco (*Nicotiana* spp.). Like many species in the family, potatoes naturally synthesize the glycoalkaloids α-solanine and α-chaconine as a mechanism of self-defense. Here again, bitter taste plays a protective role. In a study of taste perception and potato use in the Aymara people of Bolivia, Johns and Keen [115] found that growers have an elaborate vocabulary for describing potato characteristics. Two key terms are *luq’i* (bitter) and *ch’oke* (non-bitter) [115, 116]. *Luq’i* and *ch’oke* correlate with glycoalkaloid content and express whether content is too high or acceptable, and how the potatoes must be processed to make them safely edible [115].

While cassava and potatoes illustrate bitter perception’s protective functions, they are unusual in the current world. Today’s food supplies are overwhelmingly safe, and not only are bitter foods usually harmless in the quantities consumed by humans they are sometimes preferred. Such foods demonstrate that aversions driven by bitterness are not always adaptive, and can be maladaptive if they result in the rejection of viable sources of nutrition [117]. For instance, bitter melon (*Momordica charantia*) is widely used in African and Asian cuisines and consumption shows no association with disease [118]. Similarly, citrus fruits (*Citrus* spp.) are often bitter but non-toxic [119]. Rejecting these foods results in the rejection of the calories, vitamins and other nutrients they contain.

The volume and quality of modern diets make the rejection of many foods inconsequential. However, this is not always the case. The classic example of bitter perception undermining health involves cruciferous vegetables, which include common table offerings such as cabbage and Brussels sprouts. Vegetables are an essential part of the diet, yet they are chronically under-consumed worldwide [120–122]. The reasons for this are complex. However, in the case of crucifers, bitter taste is an important contributor. Crucifers utilize a defense system analogous to that of cassava in which a glucoside (sinigrin) stored by the plant is hydrolyzed by an enzyme (myrosinase) when tissue damage occurs, producing a burst of noxious compounds [123, 124]. The products of sinigrin–myrosinase reactions are easily tolerated even by children and reports of overexposure are rare. Furthermore, some products of sinigrin–myrosinase reactions have anti-cancer properties [125–127]. However, one of the products of glucosinolate–myrosinase reactions, goitrin, is intensely bitter [17]. As a result, while crucifers are an abundant and readily available source of nutrients lacking in most diets, and may even have unique advantages, they are often rejected as unpalatable [13, 15, 128, 129].

A seemingly paradoxical observation about bitterness is that it is not always aversive, and can even be attractive. For instance, tonic water, which contains quinine, is a bitter yet popular drink. Other examples include bitter vegetables such as kale, coffee, tobacco smoke and many beers. The factors driving food preferences as opposed to perception alone are complex and the basis for bitter attraction is still being unraveled [10]. However, general observations offer explanations. First, humans' cognitive abilities enable them to learn from one another, and learned information about a bitter food's being safe may override instinctive aversions. Second, humans are adept at learning through trial and error. If a bitter food is tentatively consumed without ill effects, it may be consumed again. Further, an observation offered by Reed and Knaapila is that beyond being harmless some bitter substances have rewarding effects, with caffeine and nicotine being examples, which could also drive attraction [10]. These alternatives are not mutually exclusive. In the case of coffee, for instance, people may be prompted to try it by observing its popularity, continue drinking it once it is discovered to be not only harmless but pleasurable, and eventually come to prefer it.

Another seeming paradox surrounding attractions to plant toxins is their traditional use as pharmaceuticals [130]. Myriad plant species are consumed for their healing properties, and many are noted for their bitterness, which can be a sign of potency [131, 132]. The compounds responsible are usually unknown because isolating plants' active constituents and testing their efficacy is such a challenge. Moreover, many medicines are derived from multiple species, which may contain compounds with synergistic effects. However, the specific plant compounds involved are known in a growing number of cases, and TAS2Rs responsive to them are known for a handful. The clearest example is salicin, a relative of aspirin found in willow (*Salix* spp.) that has pain killing and anti-fever properties and is an agonist of TAS2R16 [63, 133]. Others include papaverine, from poppy (*Papaver* spp.) (vasodilator; an agonist of TAS2R7, -10 and -14) and quinine (anti-malarial; agonist of multiple TAS2Rs).

Remarkably, the use of bitter plants as medicines extends to non-humans, which exhibit zoopharmognosy (animal knowledge of pharmaceuticals) [134]. Many instances of animals ingesting toxic plants for their pharmacological effects are documented [134]. The plants are frequently bitter, suggesting that taste is a signal of therapeutic value to animals just as it is for humans. In addition, the ingested plants frequently harbor compounds in classes eliciting responses from TAS2Rs, such as glycosides [134]. Some are also known to have specific health effects, such as anti-parasite activity. An especially well-documented connection between bitter taste and animal self-medication is in chimpanzees (*Pan troglodytes*) [135]. In an investigation of chimpanzee populations in Tanzania, Koshimizu *et al.* [135] found that a selectively ingested plant (*Vernonia amygdalina*) harbors bitter compounds with potent effects on intestinal parasites. The implicated compounds included sesquiterpene lactones and glucosides, which are often agonists of human TAS2Rs, implying they are also TAS2R agonists in chimpanzees. Consumption of other plants by chimpanzees have been found to reduce parasite load in the gut, and it seems likely they have similarities to *Vernonia amygdalina* with respect to taste and medicinal effect [136].

### Genetic variation and health

Potential connections between variation in bitter taste sensitivity and dietary preferences were recognized early on in taste research and continue to be the focus of much investigation [10, 137, 138]. Initial efforts to dissect taste–diet relationships took advantage of the high heritability of sensitivity to PTC and its close relative 6-*n*-propylthiouracil (PROP), which justified their use as genetic markers prior to the discovery of *TAS2R38* [137, 139–141]. Treating PTC sensitivity as a genetic marker was a successful approach, but it was constrained by several factors including the facts that PTC and PROP are synthetic and never encountered in nature, they are imperfect indicators of genotype, and they are weak predictors of variation in the perception of other compounds. Current approaches are capitalizing on genotypic data obtained directly from *TAS2R*s, which provide a more precise tool for ascertaining genetic effects on the perception of specific bitter compounds as well as foods and drinks.

The range of bitter compounds known to elicit variable taste responses due to polymorphism in *TAS2R*s is rising rapidly, particularly with respect to substances found in the diet. Most have been established using a combination of association studies in subjects and functional analyses *in vitro*. For instance, the perceived bitterness of goitrin is now known to associate with variants of *TAS2R38*, which encodes receptor isoforms exhibiting functionally divergent responses to goitrin *in vitro* [17]. Similarly, taste responses to the artificial sweeteners acesulfame K and saccharin associate with SNPs in *TAS2R31*, and responses to stevioside, a natural non-caloric sweetener, associate with SNPs in *TAS2R4* and *TAS2R14* [142–144]. Other examples include grosheimin (found in artichoke; *TAS2R19*, -*31* and -*50*), and compounds in hops (*TAS2R1*, -*14* and -*40*) [145, 146]. Quinine, an ingredient of tonic water, exhibits a notoriously complex relationship with *TAS2R*s. It activates nine *in vitro* and taste responses in subjects associate with variants in two, *TAS2R19* and -*31* [63, 147, 148]. Compounds rare in the diet have also been investigated. Perception of aristolochic acid, a food contaminant originating from *Aristolochia clematitis* in Eastern Europe, associates with polymorphism in *TAS2R43*, and salicin perception associates with variants in *TAS2R16* [102, 144, 149]. Additional substances show genotype–phenotype associations that are compelling but not yet validated such as capsaicin, piperine and ethanol (which associate with SNPs in *TAS2R3*, -*4* and -*5*) [150]. Evidence that polymorphism in *TAS2R*s shapes sensitivity to specific compounds suggests they shape the preferences for foods containing them, as well, with consequences for consumption patterns and health.

The earliest persuasive evidence for connections between genetic variation in taste, diet and health was reported by Greene in 1974 [151]. In an investigation of endemic goiter in Andean populations, Greene found that PTC sensitivity was highly variable and associated with disease, with low sensitivity associating with high susceptibility. In addition, the Andeans’ diets were rich in bitter vegetables implicated as being goitrogenic. Greene concluded that the PTC taster allele confers aversion to goitrogen containing vegetables, providing protection against their negative effects. Thus, the observed association between taste and goiter had a simple explanation. It also raised an evolutionary question: if taste sensitivity to goitrogens confers protection against exposure, why is the taster allele not fixed in the population? Greene speculated that it arises from changes in goiter susceptibility through the lifespan, with children being more susceptible than adults. Under these conditions heterozygotes with respect to the taster and non-taster alleles might have a reproductive advantage if they exhibit intermediate taste sensitivity. This would maintain both alleles in the population.

The most extensively investigated relationship between genetic variation, perception of foods and diet choices connects *TAS2R38* genotype and vegetable intake. It is well established that the perceived bitterness of vegetables, particularly cruciferous vegetables, is negatively associated with liking and intake [14]. In addition, the perceived bitterness of goitrin, a major constituent of cruciferous vegetables, associates with alleles of *TAS2R38* [17]. *TAS2R38* genotype also associates with perceived bitterness and liking of cruciferous vegetables themselves, not just goitrin [129, 152]. Further, although *TAS2R38* genotype associates with the perceived bitterness of cruciferous vegetables, it does not associate with the perceived bitterness of non-cruciferous vegetables, which is expected if goitrin content is driving the relationship [153]. The patterns are further supported by evidence that the associations are weaker for vegetables overall than for cruciferous vegetables in particular, which follows because not all vegetables are crucifers [154, 155]. Together these findings support the long-held but difficult to test hypothesis that heritable variation in bitter taste sensitivity drives preferences and consumption.

Insights into relationships between *TAS2R38* genotype, diet and health are well developed due to PTC sensitivity’s high heritability and early discovery. However, similar relationships involving other *TAS2R*s and health behaviors are being uncovered. Some involve compounds not encountered by humans until recently. For instance, variation in the perceived bitterness and intake of alcohol associates with polymorphism in *TAS2R3*, -*4*, -*5*, -*16* and -*19* as well as -*38* [150, 156–159]. Ethanol is not completely absent from the natural environment (it sometimes occurs in fermenting fruits), but it is rare and seems unlikely to be specifically targeted by TAS2Rs. A simpler explanation is that ethanol stimulates some TAS2R isoforms incidentally, imparting bitterness. Similarly, variation in *TAS2R38* associated with habitual tobacco use, another recent phenomenon [160–163]. *TAS2R38* could not have evolved under selective pressures arising from tobacco use. However, smoke contains hundreds of combustion products that could act as TAS2R agonists incidentally, driving associations. Relationships with more distant connections to health are also known, such as the association of *TAS2R16* alleles with the perceived bitterness of espresso coffee and grapefruit juice [164]. The implication of findings like these is that although variation in *TAS2R*s is a holdover from ancient processes, and rarely influences exposure to toxins encountered through foraging, it retains its importance to modern patterns of perception and behavior.

Evidence that *TAS2R*s mediate health behaviors such as alcohol and tobacco use suggests that they associate with their downstream consequences, as well. However, evidence for such effects has been elusive. To be detectable, not only does variation in bitter perception have to affect behavior, the effect of the behavior on health has to be evident amidst myriad other factors. The challenge is illustrated by patterns of association between bitter perception and body fat. The role of *TAS2R*s in shaping food preferences suggests that bitter perception is likely associated with measures such as body mass index (BMI), and such associations do exist. For instance, taste sensitivity to PROP associates with measures of body fat in some populations [165–169]. However, the associations are not universal [155, 170–172]. Moreover, while *TAS2R38* genotypes are strongly associated with taste sensitivity to PROP, which associates with BMI, associations between *TAS2R38* genotypes themselves and BMI are weak or absent [172–174]. The simplest explanation for these patterns is that PROP sensitivity is determined by factors in addition to *TAS2R38* genotype and it is overall taste sensitivity, not *TAS2R38*-mediated sensitivity alone, that drives the associations [175].

Here again, it is important to note the potential importance of extraoral TAS2Rs. The roles of *TAS2R*s in hormonal responses and bronchodilation in the gut and lungs, imply that genetic variation in *TAS2R*s could exert health effects via those mechanisms. Findings from genotype-phenotype association studies targeting *TAS2R*s emphasize their potential importance. Consistent with evidence that ingestion of bitter compounds can produce hormonal responses in gut cells, Dotson *et al.* [176] found that variation in *TAS2R9* associates with glucose dysregulation and Clark *et al*. [177] found that variation in TAS2R42 associates with thyroid hormone levels. Similarly, in an investigation of variation of associations between TAS2R38 variants and respiratory health, Lee *et al*. [178] found that variation in *TAS2R38* associates with susceptibility to infection.

It is important to recognize that the elusiveness of direct associations between genotypic variation in *TAS2R*s and complex health measures such as BMI does not imply that they are unimportant. First, associations between variation in *TAS2R*s and behaviors such as vegetable consumption and tobacco use are consistent. These behaviors have health impacts, and understanding the role of *TAS2R*s in shaping them will reveal strategies for modifying them, improving health outcomes. Second, even if associations between *TAS2R* variation and complex traits such as BMI are weak statistically, they are potentially important on population scales, which are the primary focus of public health. That is, effects so small they are difficult to detect in samples of hundreds or thousands of subjects could be large enough to merit intervention in populations composed of hundreds of thousands or millions of individuals.

## CONCLUSIONS

Insights into the evolutionary processes underlying bitter taste perception continue to shed light on the complex connections between ancient evolutionary processes and modern human health. They also continue to expand. While early studies focused on single *TAS2R* loci, and then on *TAS2R* repertoires in select taxa, developments in genomics are accelerating the pace at which data are emerging. Whole-genome sequencing, in particular, is providing a basis for comparing *TAS2R* genes and gene repertoires within and across species at high resolution. As the focal point of biomedical and health research, human data are being generated at an especially high rate. This is fostering population genetic research with a global outlook and association analyses in the largest phenotyped cohorts studied to date, offering opportunities for research on new scales.

The power of coming evolutionary studies is illustrated by the Vertebrate Genomes Project (VGP), an initiative to sequence the complete genomes of ∼70 000 vertebrate species (vertebrategenomesproject.org) [179]. The VGP and related efforts are providing access to data not only from large numbers of species but from species outside the research mainstream, which typically focuses on mammals, especially primates. Gene birth–death processes are a particularly promising line of research. Given that the number of functional *TAS2R*s varies from 0 to more than 100 across species it is clear that birth–death processes are an essential feature of TAS2Rs' evolution, yet little is known about them. Genomic data from novel species also provide inventories of homologous but functionally divergent TAS2R isoforms, which are a resource in efforts to dissect the molecular basis of receptor response. Similarly, large-scale resequencing projects within humans, which now extend beyond the 1000 Genomes Project, are providing genetic portraits of global populations at unprecedented levels of detail.

Like evolutionary studies, efforts to define taste–health connections are capitalizing on the advent of genomic technologies. While early efforts were limited to tens or hundreds of subjects, the economics of data collection are producing datasets composed of tens of thousands or even hundreds of thousands of subjects [96, 180, 181]. Data on this scale offer high statistical power, making them a promising resource for resolving relationships between *TAS2R* polymorphism and health traits. They are also potentially able to incorporate evolutionary evidence for the functional importance of specific sites to make prior predictions about phenotypic effects. However, most large scale studies to date have focused on associations between genome-wide markers and health traits generally, rather than on *TAS2R*s and taste phenotypes specifically. An essential step moving forward will be the establishment of cohorts with phenotyping components directly aimed at sensory phenotypes, behaviors and outcomes.

**Conflict of interest:** None declared.
